# Intramolecular Annulation of Gossypol by Laccase to Produce Safe Cottonseed Protein

**DOI:** 10.3389/fchem.2020.583176

**Published:** 2020-12-01

**Authors:** Lin Wang, Ming Chen, Xuecai Luo, Yanan Fan, Zai Zheng, Zongqin He, Ruochun Yin, Tao Meng, Shuyang Xu, Yu Pan, Jihu Su, Jiangfeng Du, Liang Zhang, Xiaohe Tian, Yupeng Tian, Dongdong Chen, Honghua Ge, Nannan Zhang, Ping Li

**Affiliations:** ^1^Research Center for Translational Medicine at Shanghai East Hospital, School of Life Sciences and Technology, Tongji University, Shanghai, China; ^2^School of Life Sciences, Anhui University, Hefei, China; ^3^Chinese Academy of Sciences Key Laboratory of Microscale Magnetic Resonance, Department of Modern Physics, University of Science and Technology of China, Hefei, China; ^4^National Engineering Laboratory for Cereal Fermentation Technology, Jiangnan University, Wuxi, China; ^5^College of Chemistry and Chemical Engineering, Anhui University, Hefei, China

**Keywords:** gossypol, laccase, cottonseed protein, catalyzes intramolecular annulation, food industry

## Abstract

The presence of the phenol gossypol has severely limited the utilization of cottonseed meal and oil in the food and animal feed industries. Highly efficient means of biodegradation of gossypol and an understanding of the cytotoxicity of its degradation products remain outside current knowledge and are of universal interest. In this work, we showed for the first time that laccase can catalyze the intramolecular annulation of the aldehyde and hydroxyl groups of gossypol for the *o*-semiquinone radical and originate the released ·OH radical. It was further found that the oxidation of aldehyde groups significantly decreases reproductive toxicity and hepatotoxicity. These results indicate a novel detoxification pathway for gossypol and reveal the crucial role played by radical species in cyclization. This discovery could facilitate the development of safe, convenient, and low-cost industrial methods for the detoxification of cotton protein and oil resources.

## Introduction

Cotton (*Gossypium*) is a highly important agricultural crop all over the world. It provides two main products: cotton fiber and cottonseed. The annual report of the USDA indicates that the global yield of cotton and cottonseed in 2015–2016 was ~24 and 42 million tons, respectively. The former is generally used as a raw material for the textile industry, and the latter supplies cottonseed oil and cakes for the food and animal feed industries. Annual global cottonseed production could provide enough protein to meet the annual dietary needs of half a billion people (Adams et al., 1960; Sunilkumar et al., 2006). However, gossypol is present at high concentrations in the pigment glands of cottonseed plants, and its ingestion subjects mammals to several sustained injuries, including reduction of the blood's oxygen-carrying capacity, genital abnormalities such as impotence and low sperm count, and lysine deficiency (Adams et al., 1960; Sunilkumar et al., 2006; Câmara et al., 2015). This cytotoxicity arises from aldehyde groups in the gossypol molecule, and the polyphenolic moiety is pharmacologically active (Dodou et al., 2005; Long et al., 2009; Yang et al., 2012). Therefore, to meet the demand for cottonseed products and therapeutic agents with aldehyde-free gossypol, a safe means of detoxifying gossypol-containing cotton is urgently required. Several methods of achieving this have been developed. The biodegradation of gossypol by the fungal channeling of metabolites appears promising, but it is still under development (Baugher and Campbell, 1969; Weng and Sun, 2006). Gossypol's structural similarity to naphthalene derivatives allows the catabolic pathway of polycyclic aromatic hydrocarbons (PAHs) to be mimicked to examine how its aromatic ring can cleave to CO_2_, initiated by monooxygenases (as occurs in fungi and insects) (Mao et al., 2007). While this method is available, the complexity of the metabolic pathways involved is a notable stumbling block. Gene engineering is another possibility, although it is quite costly, and interfering with gossypol production in this way may adversely affect cotton yield (Bottger et al., 1964; Jenkins et al., 1966; Li et al., 2002; Sunilkumar et al., 2006; Palle et al., 2013). Laccase (benzenediol: oxygen oxidoreductase, EC1.10.3.2) has long been associated with a range of reactions, including oxidative coupling, Michael additions, elimination reactions, quinone formation, and aromatic compound polymerization, which occurs by abstracting an electron from a substrate to produce a free radical and reducing oxygen to water (Witayakran and Arthur, 2009). Laccase and the so-called dirigent proteins are responsible for biaryl synthesis, including the coupling of hemigossypol (Davin et al., 1997; Wei et al., 2012; Effenberger et al., 2015). By contrast, laccase from *Pleurotus florida* may also guide gossypol degradation, but this mechanism requires further detailed investigation (Rajarathnam et al., 2001).

Bearing all of this in mind, we demonstrated that the reaction of intramolecular annulation between the aldehydic and hydroxyl groups of the gossypol molecule is catalyzed by laccase. By combining nuclear magnetic resonance (NMR) spectroscopy, electron spin resonance (ESR) spectroscopy, and molecular simulation, we demonstrated that laccase is capable of catalyzing the intramolecular annulation of the aldehyde and hydroxyl groups of gossypol on the o-semiquinone radical and to originate the released ·OH radical. Furthermore, an *in vivo* mouse model was used to validate the proposal that the enzymatic method could significantly decrease the toxicity of gossypol, producing much less hepatotoxicity and reproductive toxicity. This novel methodology not only introduced an alternative gossypol detoxification pathway but also shed light on inexpensive industrial methods for the detoxification of cotton protein and oil resources with a promising degree of safety.

## Results and Discussion

**Figure 1:**
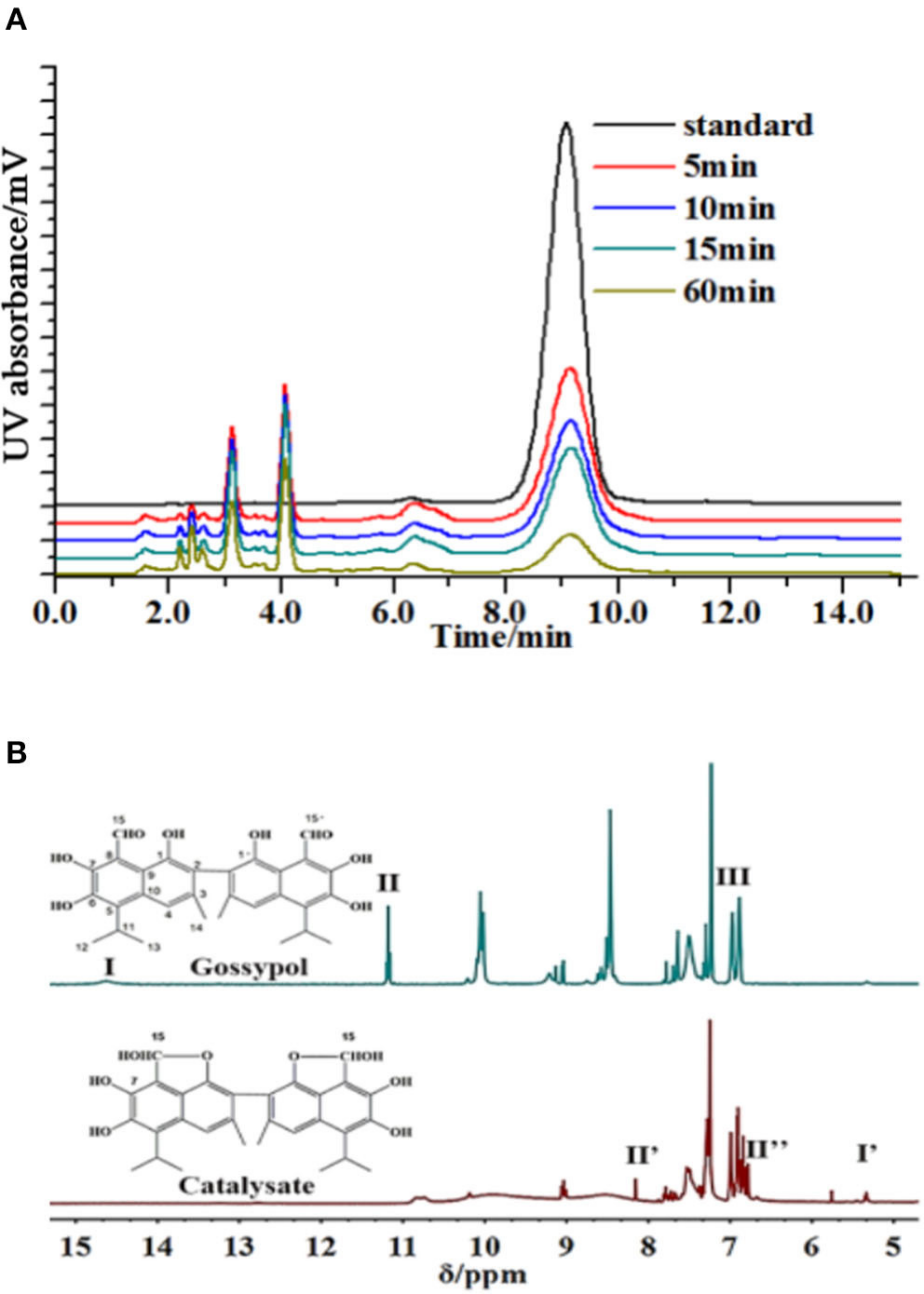
Degradation of gossypol by laccase. **(A)** Gossypol (0.2 mg/ml) with laccase (0.5 U/ml) and catalysates at 10 ml, 30°C, pH 6.5, for 5, 10, 15, and 60 min, analyzed by HPLC. The degradation rates of 5, 10, 15, and 60 min were 86.09, 89.35, 90.11, and 95.86%, and the majority of substrate removal occurred over the first 5 min, and then the substrate removal was kept at a high level. **(B)** ^1^H NMR spectra of gossypol and products (initial concentration was 0.2 mg/ml) without substrate in DMSO-d6. Signals indicated with Roman numerals are absent or present in reaction samples.

The produced intermediates were characterized. It was shown that gossypol was effectively decomposed, and a series of intermediates appeared when laccase (*Trametes versicolor*) was added (Figure 1A). In the filtered solution, the rate of gossypol elimination was proportional to the enzyme activity and cultivation time. The retention time of compound 1 differed from that of the standard, although it had identical molecular weights (Supplementary Figure 1). Correspondingly, three new signals (I′ = 7,7′-OH, II′ = 15,15′-OH, and II″ = 15,15′-H) emerged, and 1,1′-hydroxyl (III), 7,7′-OH (I), and 15,15′-aldehyde group (II) disappeared in the ^1^H NMR spectrum of the reaction mixture without a substrate (Figure 1B, Supplementary Figures 2–5). We assigned the C15, 1 and C15′, 1′ centers of compound 1 to bind the aldehyde group with the hydroxyl and produce the furan ring, but the structures of the residual intermediates remained unknown (Supplementary Figures 6, 7).

**Figure 2:**
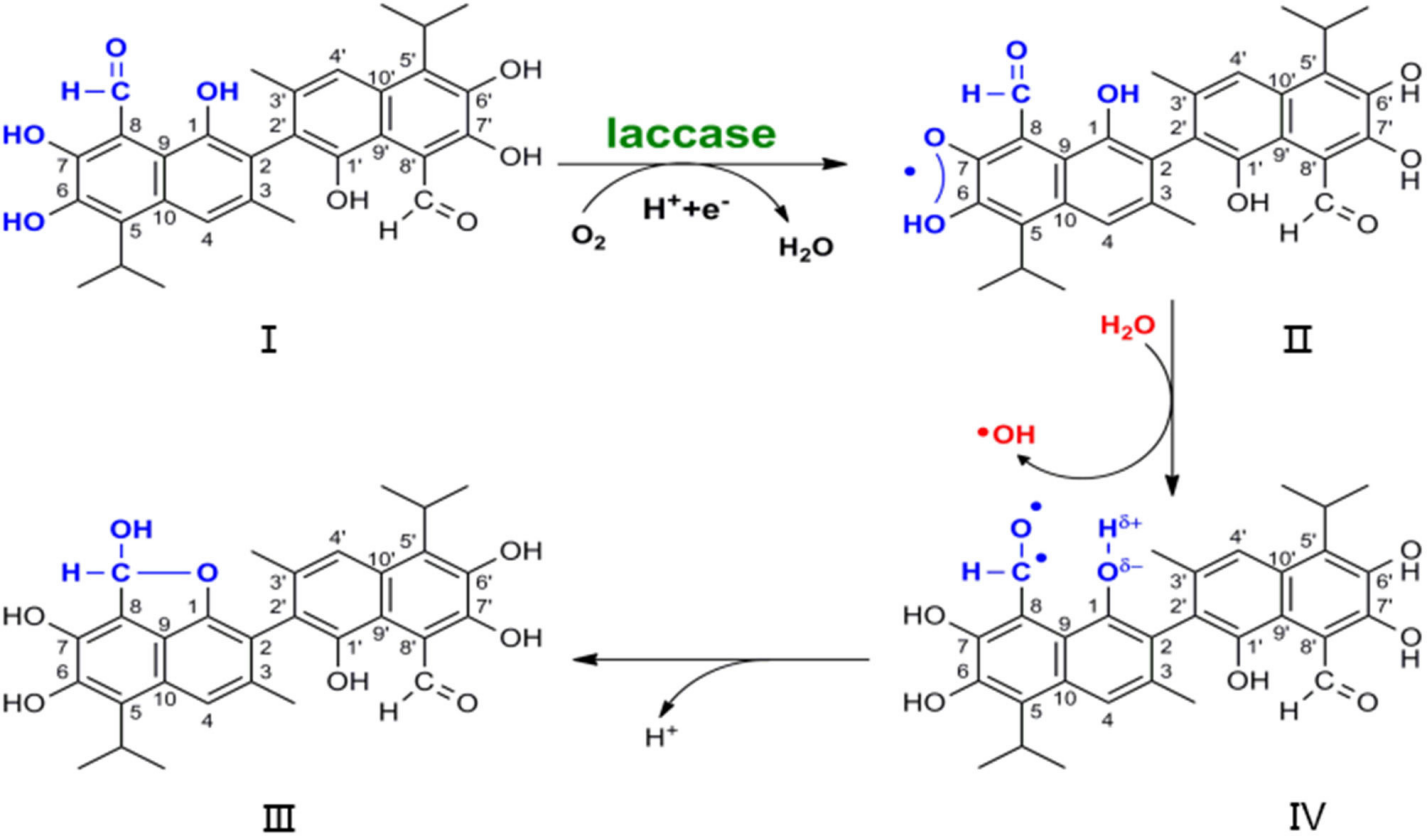
Scheme of nuclear magnetic resonance in the development from substrate I to product IV, which earthly conjecture the degradation of gossypol by laccase.

Various tautomerisms have previously been observed in NMR and X-ray analyses of gossypol conformation, but the radical trigger property of dimethyl sulfoxide (DMSO) in atmosphere obviates the discussion of the dilactol form tautomer (Adams et al., 1960; Ibragimov et al., 1992; Kenar, 2006). Oxy-radicals have been trapped upon exposure of DMSO to air, without radiation input (Supplementary Figure 8) (Doorslaer et al., 1999; Jerzykiewicz et al., 2011). Hence, the formation of **I** to **IV**, as shown in Figure 2, is not a physical process but a reaction. Free radicals play a vital role in the development process of substrate **I** to product **IV**, resulting in hydroxy-aldehyde condensation for the hydroxyl group at position 1 and for the aldehyde group at position 8. The lactol tautomer that was previously observed may be a product that is initiated by the radical (Ibragimov et al., 1992; Kenar, 2006). Polyphenols and benzofuran moieties are abundant in natural molecules that possess potent physiological activity (Montamat et al., 1982; Long et al., 2009). Moreover, understanding their construction pattern has been the aim of much research (Rafi et al., 1985; Vosburg et al., 2002; Tranchimand et al., 2006; Witayakran and Arthur, 2009; Effenberger et al., 2015).

**Figure 3:**
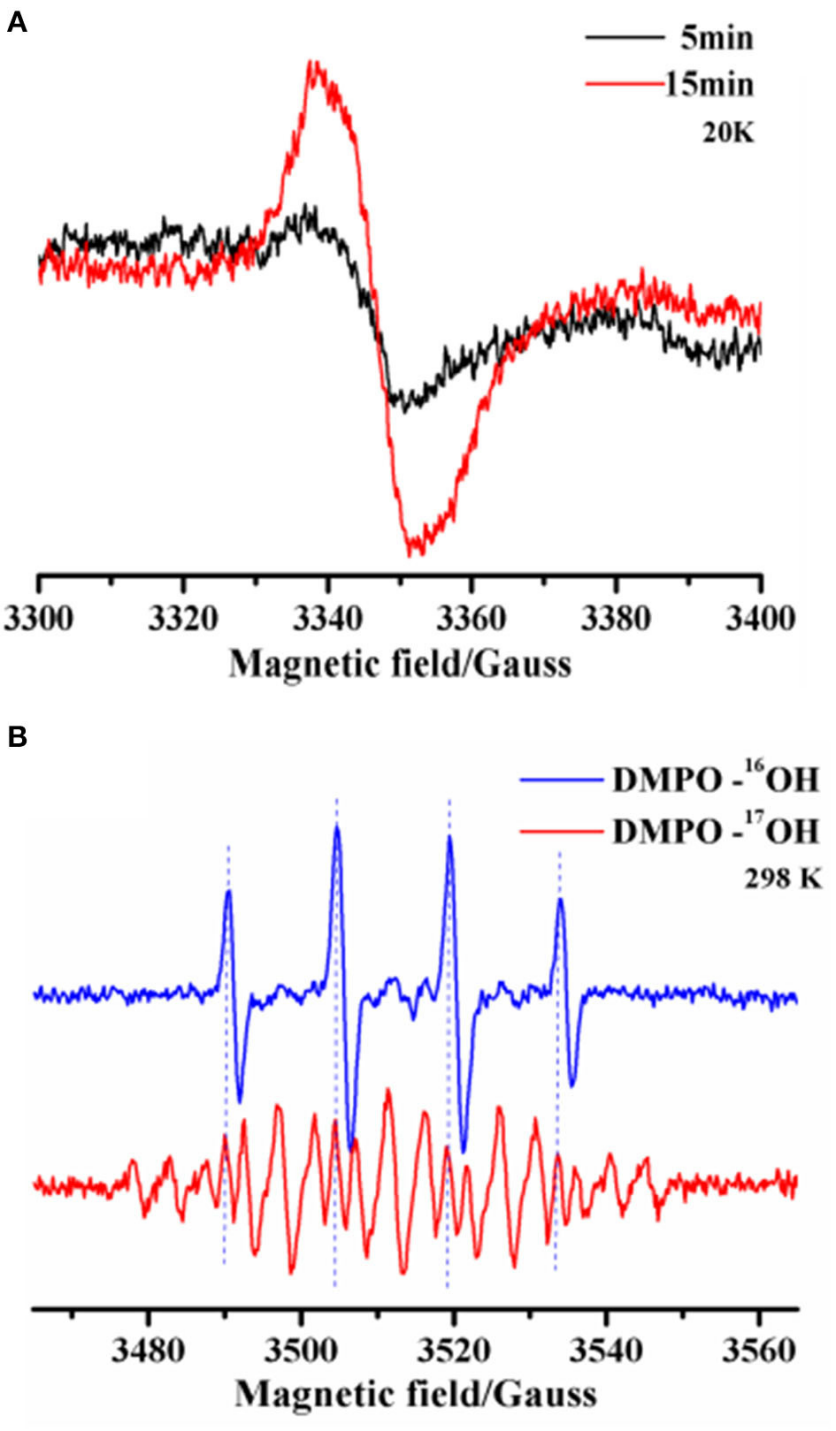
**(A)** X-band (9.39 GHz) ESR spectra were recorded to monitor metastable radicals for the laccase-catalyzed oxidation of gossypol trapped by freezing. The reaction was allowed to proceed at room temperature for 5 and 15 min, and then the solutions were quickly stored using liquid nitrogen, where they were kept until ESR measurements could be performed. **(B)** Room temperature X-band (9.86 GHz) ESR spectra and the simulation of DMPO adduct radicals after 5 min of laccase-catalyzed oxidation in the normal (${\text{H}}_{2}^{16}$O) or 17O-labeled (${\text{H}}_{2}^{17}$O, ~80% atom % ^17^O) aqueous solution. The legend includes DMPO-16OH and DMPO-^17^OH adduct radicals.

Next, we performed ESR analyses at 20K to identify intramolecular annulation. Time-course freeze-trapping experiments showed that the original product was an o-benzosemiquinone radical with a symmetrical feature at g-2.004 showing a peak-to-trough width of 7-G (Figure 3A). Previous research has shown that type 1 copper binds to o-hydroxyl (Quintanar et al., 2007). Within 5 min, the o-benzosemiquinone radical was consumed after aldehyde group activation, and the corresponding bonds were formed. A cumulative radical signal was observed on a longer time scale, compatible with a decrease of aldehyde groups in gossypol (Figure 1A).

We also used a room-temperature spin-trapping protocol to trap the hydroxyl radical *in situ*. The isotropic HFCs of the α-nitrogen and β-proton, characterized by a_N_ = a_Hβ_ = 14.8 G, led to a four-line (1:2:2:1) spectrum. This is a typical characteristic of the ·OH radical. ^17^O isotopic-labeled solvent water (${\text{H}}_{2}^{17}$O, ~80% atom % ^17^O) was used to indicate the origin of the ·OH radical. The four-line ESR signal of the DMPO (5,5-Dimethyl-1-Pyrroline-N-Oxide)-·^16^OH radical (^16^O, 99.757% natural abundance) was replaced by a weaker signal due to the further hyperfine splitting of ^17^O (I = 5/2) in the DMPO-·^17^OH radical (a_N_ = a_Hβ_ = 14.8 G, and a_17O_ = 4.6 G) (Figure 3B). Moreover, the removal of the DMPO-·^16^OH radical signal by the ^17^O isotope unambiguously precluded other oxygen radicals during the process. Although laccase reduces oxygen to water in the trinuclear copper center, water is another substrate of the oxidation, which is a consequence of the laccase-initiated radical process (Shinde et al., 2009; Chen et al., 2017). The fact that everlasting radicals led to a series of non-enzymatic chain reactions could be helpful for discovering the reason for the broad substrate selectivity of laccase. Thus, we demonstrated that laccase-initiated o-benzosemiquinone radicals facilitate the activation of the aldehyde group and stepwise aldol condensation, without the participation of an o-hydroxyl.

**Figure 4:**
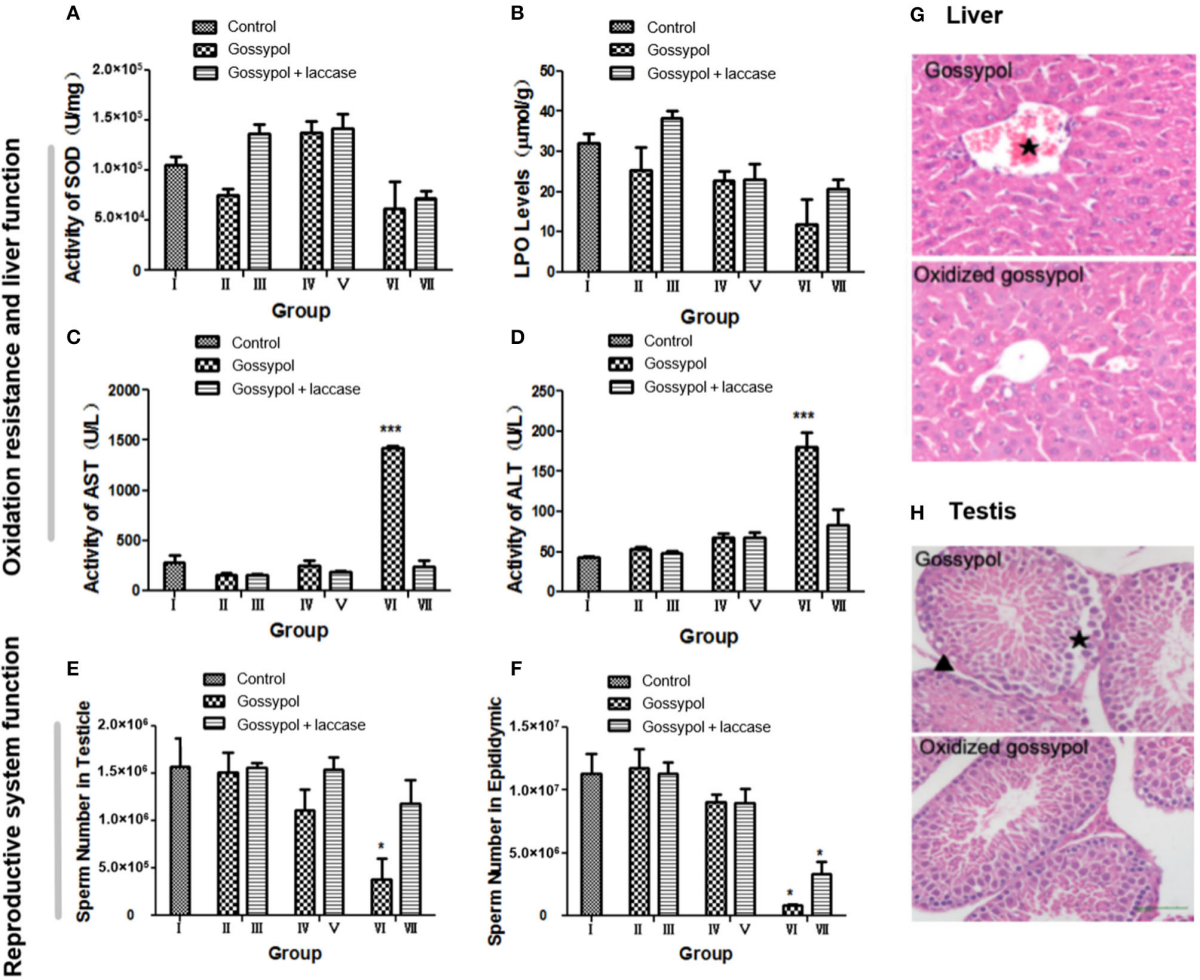
Experimental results for the toxicity of gossypol and its oxidation products to mice. **(A)** The activity of SOD in each group: the value for the group treated with 100 mg/kg was larger than for other groups. The value was also higher in groups with laccase between the groups with same gossypol dosage. **(B)** The LPO value for each group decreased with increasing dosage and was higher in groups with laccase compared to the groups with the same gossypol dosage. **(C)** AST activities: those for group V were apparently higher than the control, while others showed no significant difference. **(D)** ALT activities were higher in the experimental group than in the control and grew with dosage, with a maximum in group V. In the group treated with 200 mg/kg, the activities of AST and ALT significantly increased with gossypol only, with a return to normal in the groups with laccase. **(E)** The numbers of sperm in the testicles showed a decline with increased gossypol dosage, and the numbers were higher in the groups with laccase between groups with the same gossypol concentration. **(F)** The numbers of sperm in the epididymis markedly decreased with increased gossypol dosages. **(G)** Hematoxylin–eosin-stained sections of mouse liver (original magnification ×400). **(H)** Hematoxylin–eosin-stained sections of mice testis (original magnification ×400). *p < 0.05, ***p < 0.001, n = 8, ⋆central vein, ▴inflammatory cells.

The safety of the residual reaction mixture should be confirmed before this method is made industrially available. To investigate whether gossypol transformation produces other toxic derivatives, we performed a toxicology test of the results on male mice. The groups of mice were as follows: I, mice treated with 10% CMC-Na; II, mice treated with gossypol (50 mg/kg body weight [BW]); III, mice treated with gossypol (50 mg/kg BW) catalyzed by laccase; IV, mice treated with gossypol (100 mg/kg BW); V, mice treated with gossypol (100 mg/kg BW) catalyzed by laccase; VI, mice treated with gossypol (200 mg/kg BW); VII, mice treated with gossypol (200 mg/kg BW) catalyzed by laccase. Gossypol, a derivative of aromatic aldehydes, is an antioxidant. Liver damage was translated from functional damage to organic damage as the gossypol dose increased, which induced an irregular change in SOD (Figure 4A). Gossypol inhibits the generation of lipid peroxides, but this effect was reversed by laccase (Figure 4B). The value of lipid peroxides was higher in group III than other groups. One reason for this could be that the LPO level was positively related to free radicals whose formation was related to gossypol concentration with a certain laccase. Active metabolites such as gossypol will break the dynamic balance of hepatocyte oxidation and anti-oxidation after entering the mouse body, the SOD and LPO content in the cell fluctuates, and SOD and LPO are key enzymes involved in liver detoxification. If the content of SOD and LPO in the organism is not stable, ROS will accumulate and cause oxidative damage to cells and tissues (Park et al., 2020). When these toxic substances are pre-oxidized *in vitro*, the metabolic activity of the product is reduced (Alharbi et al., 2019), so that the content of SOD, LPO, and ROS in liver tends to be stable. Laccase was found to partly heal the liver damage caused by gossypol (Figures 4C,D). Liver function was found to be in the normal range at the low (groups II and III) and moderate (groups IV and V) doses, but a difference was found at high (groups VI and VII) doses. The liver was seriously damaged in group VI mice, shown by the significantly increased activities of AST and ALT, but it was normal in group VII. The sperm number shown in Figures 4E,F indicates that gossypol has a negative effect on mice sperm that is more significant with larger doses and is more obvious in the epididymis than in the testicle. However, the sperm-killing effects of gossypol were restricted after it was catalyzed by laccase, and they were much clearer in the highest-dose group. It has been reported that gossypol causes severe hepatocyte damage in addition to abnormal localization of the hepatocytic nuclei (El-Sharaky et al., 2010). Histological analyses of the testicular and hepatic tissues have indicated that laccase marginally heals mice from organic damage (El-Sharaky et al., 2009). As shown in Figure 4G, after administration of gossypol at 50 mg/kg BW, liver cells were disordered, swollen, and without their basic outline visible. Damage to the central venous congestion was particularly strongly manifested, and a large number of inflammatory cells appeared. A reducing trend occurred in the groups where gossypol was administered with laccase. This indicates that laccase could reduce gossypol toxicity to the liver. Moreover, gossypol is well-known for its toxic effects to male reproductive organs. It can interfere with reproduction, damage sperm, disrupt the estrous cycle, and produce embryonic lethality (Câmara et al., 2015; Duarte Júnior et al., 2018; Luz et al., 2018). Our results found that mice exposed to gossypol at 100 mg/kg BW endured partially disrupted basal membranes, exfoliation of spermatogonia, and the appearance of intraepithelial vacuoles in the testis, but this damage was relieved in mice who were administered oxidative annulation products (Figure 4H). This indicates that the degradation products produced by laccase reduced reproductive toxicity in mice. This remission may explain the hypothesis that gossypol toxicity is conquered to some extent due to laccase conversion of aldehyde groups to cyclic acetals. Compared to control groups with similar doses of gossypol, the mice given the laccase intramolecular annulation products maintained higher rates of BW gain, showed higher testicular sperm counts, and exhibited improved responses to organic injury, particularly at high doses (Supplementary Figures 9–11), which reflects that laccase abated the toxicity of gossypol. This implies that it may be possible to use gossypol derivatives to combat cancer or viruses with reduced side effects (Montamat et al., 1982; Wang et al., 2008).

## Conclusions

Biotoxins, including gossypol, pose a persistent challenge for the food and animal feed industries. Here, we show that laccase has the capacity for intramolecular cyclization via radicals and water to produce hydroxyl radicals. These results are a contribution to the exploration of regioselective catalysis. This methodology has potential as a safe detoxifier for gossypol in cottonseed protein and oil. Industrial-scale production of laccase can lead to the increased production of usable cotton protein and oil, and these may be used to sustain ever-increasing populations.

## Data Availability Statement

The original contributions generated for this study are included in the article/Supplementary Materials, further inquiries can be directed to the corresponding author/s.

## Ethics Statement

The animal study was reviewed and approved by Anhui University Animal Care Committee.

## Author Contributions

All authors listed have made a substantial, direct and intellectual contribution to the work, and approved it for publication.

## Conflict of Interest

The authors declare that the research was conducted in the absence of any commercial or financial relationships that could be construed as a potential conflict of interest.
