# Supplementary material for: Intramolecular Annulation of Gossypol by Laccase to Produce Safe Cottonseed Protein
Source: Front Chem. 2020 Dec 1;8:583176. doi: 10.3389/fchem.2020.583176 (PMC7736553; doi:10.3389/fchem.2020.583176)
Supplement: Supplementary file 1 [file Table_1.DOCX]

**Intramolecular annulation of gossypol by laccase for safe cottonseed protein**

Lin Wang^a,b^, Ming Chen^c^, Xuecai Luo^b^, Yanan Fan^b^, Zai Zheng^b^, Zongqin He^b^, Ruochun Yin*^b^, Tao Meng^b^, Shuyang Xu^b^, Yu Pan^b^, Jihu Su^c^, Jiangfeng Du^c^, Liang Zhang^d^, Xiaohe Tian^b^, Yupeng Tian^e^, Dongdong Chen^b^, Honghua Ge^b^, Nannan Zhang^b^, Ping Li*^a^

^a^School of Life Sciences and technology, Tongji University, Shanghai, 200092, China.

^b^School of Life Sciences, Anhui University, Hefei, Anhui 230601, China.

^c^CAS Key Laboratory of Microscale Magnetic Resonance, and Department of Modern Physics, University of Science and Technology of China, Hefei 230026, China.

^d^National Engineering Laboratory for Cereal Fermentation Technology, Jiangnan University, Wuxi, 214122, China.

^e^College of Chemistry and Chemical Engineering, Anhui University, Hefei, Anhui 230601, China

^*^Correspondence to: rcyin@ahu.edu.cn, liping01@tongji.edu.cn

† These authors contributed equally.

*Correspondence to: [rcyin@ahu.edu.cn](#mailto:rcyin@ahu.edu.cn) , [sujihu@ustc.edu.cn](#mailto:sujihu@ustc.edu.cn)

[Materials 3](#_Toc1673)

[Animals 3](#_Toc15860)

[HPLC 3](#_Toc20991)

[LC-MS measurements 3](#_Toc19243)

[Freeze-trapped EPR measurements at liquid helium temperature 4](#_Toc21293)

[The spin-trapping EPR measurements at room temperature 4](#_Toc26182)

[IR measurements 4](#_Toc23181)

[NMR spectroscopy 4](#_Toc10287)

[Catalysates preparation of oxidative gossypol catalyzed by Laccase for mice test 4](#_Toc26198)

[Liver Function and Oxidation Resistance Test assay 4](#_Toc879)

[Sperm counts 5](#_Toc32617)

[Histopathological analysis 5](#_Toc17367)

[Statistical analysis 5](#_Toc30442)

[Figure S1 5](#_Toc8267)

[Figure S2. 6](#_Toc20814)

[Figure S3. 6](#_Toc6145)

[Figure S4. 6](#_Toc21283)

[Figure S5. 7](#_Toc24251)

[Figure S6. 7](#_Toc1253)

[Figure S7. 7](#_Toc30657)

[Figure S8. 8](#_Toc28307)

[Figure S9. 8](#_Toc23965)

[Figure S10. 9](#_Toc4329)

[Figure S11. 10](#_Toc17656)

# Materials

Standard chemicals and authentic compounds, including Gossypol (purity ≥ 95%), Laccase (*Trametes versicolor,* purchased from Sigma-Aldrich, St. Louis, 0.5 U/mg, Unit Definition: One unit corresponds to the amount of enzyme which converts 1μmol of catechol per minute at pH 5.0 and 25℃). 5,5-Dimethyl-1-Pyrroline-N-Oxide (DMPO) was from Dojindo International. Residual chemicals were from Sinopharm unless specified otherwise.

# Animals

Male Chinese Kunming mice (27.1±3.4 g) used in this study were obtained from the Experimental Animal Center of Anhui Medical University (Hefei, China). Animals were housed in cages with a maximum of eight mice per cage at a constant temperature of 23℃ with 12 h light/dark cycle. The mice had free access to standard rodent chow and water ad libitum throughout the study duration. The experimental protocols were approved by the Committee on the Ethics of Animal Experiments of the Anhui University.

# HPLC

The catalysis was performed at 30℃ containing 0.5 U/mL laccase and 0.2 mg/mL gossypol for 10 mL reaction fluid during 0-60 minutes, respectively, and extracted by ethyl acetate (4℃). Samples were then separated by reversed-phase HPLC, analyzed, and peak identities were confirmed by LC-MS as described below.

HPLC samples were analyzed on a Hitachi L-2000 system using an Agilent Eclipse XDB-C18 column (5 μm, 4.6 x 150 mm). Using distilled water with 0.05% phosphoric acid and acetonitrile (80/20, v/v) as a mobile phase at a flow rate of 0.8 mL/min to separated the samples. Elution of metabolites was monitored at 238 nm by an UV detector. Retention time and UV spectra were compared to those of standard samples of gossypol.

# LC-MS measurements

Gossypol and catalysate were analyzed using liquid chromatography coupled mass spectrometry (LC-MS). The samples were separated by a HPLC system equipped with the same C18 reverse-phase column as above, using distilled water with 0.05% formic acid and acetonitrile (80/20, v/v) as a mobile phase at a flow rate of 0.5 mL/min. The metabolites were first confirmed by the retention time and UV absorption at 238 nm. For MS analysis, a quadrupole tandem mass spectrometer (Thermo Scientific LTQ-Orbitrap XL system) outfitted with an electrospray ion source (ESI-MS) was used. In the MS scan mode, scans were carried out between m/z^-^ values of 100 and 1500.

# Freeze-trapped EPR measurements at liquid helium temperature

The freeze-trapped electron paramagnetic resonance (EPR) spectra are performed with a Bruker EMX EPR spectrometer. The EPR settings are: modulation amplitude, 2 gauss; microwave power, 2 mW; frequency, 9.39 GHz; temperature, 20 K.

The experimental solutions (200 μL) is sampled and then frozen quickly at liquid nitrogen after 5 or 15 minute’s reaction at room temperature, and stored at liquid nitrogen until the EPR measurement.

# The spin-trapping EPR measurements at room temperature

The mixture solution has laccase (0.5 U/mL), gossypol (0.25 mg/mL), DMPO (200 mM/L). The reaction is undergoing at 30℃, 10 mL about 3~4 min, then trapping reagent DMPO is quickly added to trap the radicals for EPR measurements.The EPR settings of the X-bandBrukerA300 EPR spectrometer are: modulation amplitude, 2.0 gauss; microwave power, 5.32 mW; frequency, 9.86 GHz; room temperature.

# IR measurements

To collect the intermediates, the catalyzed reaction was stopped after 30 min, extracted by ethyl acetate (4 ℃). After removal of residual gossypol by macroporous resin-HPD400, the crude extraction was subjected to Infrared spectroscopic (Vertex80+Hyperion2000) measurements.

# NMR spectroscopy

Sample was prepared as “IR measurements” and tested in DMSO (Dimethyl sulfoxide)-*d*_6_, using TMS as an internal standard at 298K (400MHz/AVANCE II).

# Catalysates preparation of oxidative gossypol catalyzed by Laccase for mice test

The catalysis solution contains laccase (0.5 U/mL), gossypol (1 mg/mL), and the reaction was carried out at 30℃ with 120 rpm stirring for 2 hours. The culture was freeze-dried and re-dissolved in anhydrous alcohol. The sediment was then separated from the media by centrifugation (5000 rpm, 10 min), extracts were evaporated to dryness and dissolved in 10% Carboxymethyl Cellulose sodium (CMC-Na). The culture was divided into three doses with different concentrations in which the initial gossypol concentration were 50 mg/kg body weight (BW), 100 mg/kg BW, 200 mg/kg BW, individually.

# Liver Function and Oxidation Resistance Test assay

The activities of Aspertate Aminotransferase (AST) and Alanine transaminase (ALT) in serum were determined by Adicon Clinical Laboratories.INC (Hefei, China). The activity of Superoxide Dismutase (SOD) and the level of Lipid Peroxide (LPO) in liver homogenate were measured by radioimmunoassay using commercial kit as per manufacturer's instruction (Nanjing Jiancheng Bioengineering Institute, China).

# Sperm counts

At necropsy, spermatozoa were obtained from testis and epididymis of each mouse in physiological saline maintained at 37℃, and their number was assessed. For assessment of sperm number, a drop of sperm suspension was smeared on a blood cell count plate and observed under microscope.

# Histopathological analysis

For histological studies, testis randomly selected from left or right sides of mice and liver were fixed in formalin, dehydrated in graded ethanol series, cleared in benzene and embedded in paraffin. Tissues were sectioned, and the sections were cut and stained with hematoxylin and eosin (H&E).

# Statistical analysis

All assays were performed three times independently, with one representative experiment shown. Data were expressed as mean ± standard deviation(SD). Statistical analysis was performed using GraphPad Prism 5.0 (GraphPad Software Inc., San Diego, CA, USA). One-way ANOVA, followed by the Tukey post-hoc test was used to analyze the statistical significance among multiple groups. P-values<0.05 were considered statistically significant.


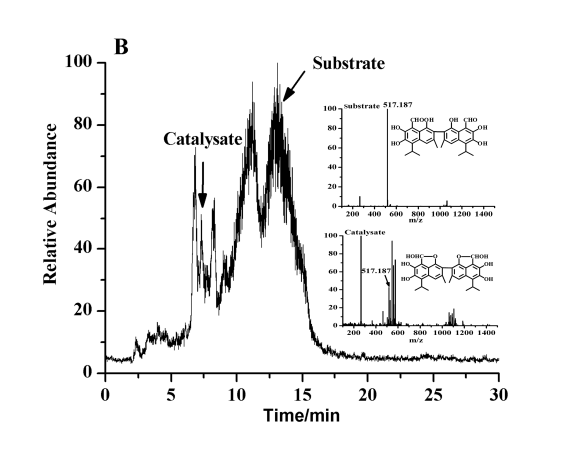

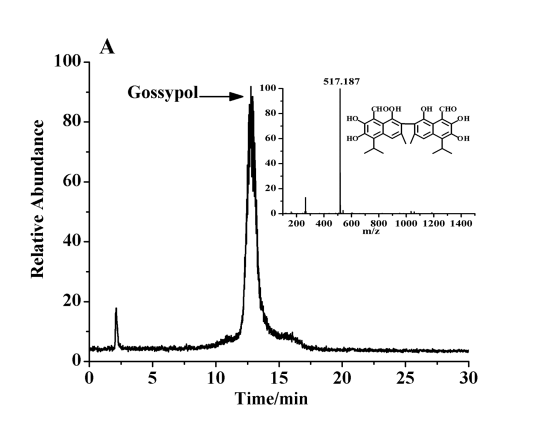


Figure S1. HPLC-MS spectrum of gossypol and catalysate. (A) Gossypol (0.2 mg/mL，present at 12:05). (B) Catalysate of gossypol by laccase (0.2 mg/mL for initial gossypol concerntion, position in 7:27). The MS data were obtained by LC-MS online analysis as described in Experimental Procedures. The mass spectrometer was operated in negative ion mode. Both compounds showed the same m/z^-^ of 517.187 as the gossypol standard.


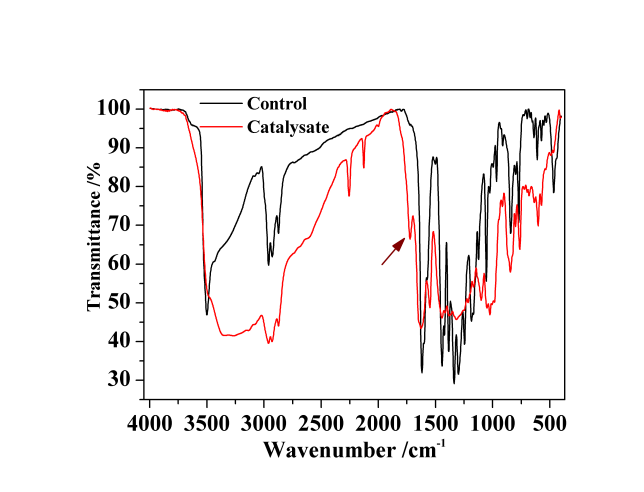


Figure S2. IR (KBr disk) spectrum analysis. The strong absorption at 1722.3 cm^-1^ (indicated by the arrow) was hinted to the C=C double bond stretching mode in the furan ring in catalysate.


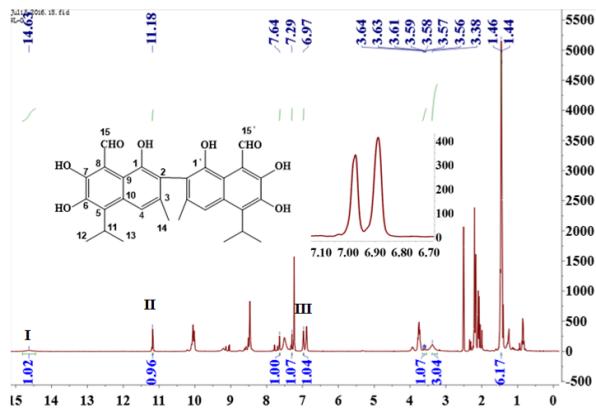


Figure S3. ^1^H NMR spectrum of gossypol. The spectrum was recorded at room temperature in DOMS-d6 using TMS as an internal standard reported in δunits. ^1^H NMR (400 MHz, DMSO-d6) δH 14.63 (s, 7,7’-OH), 11.18 (s, CHO-15,15’), 7.64(s, H-4,4’), 7.29(s, 6,6’-OH), 6.97(s, 1,1’-OH), 3.59(m, H-11,11’), 3.38(d, H-14,14’), 1.46(d,H-12,12’,H-13,13’).


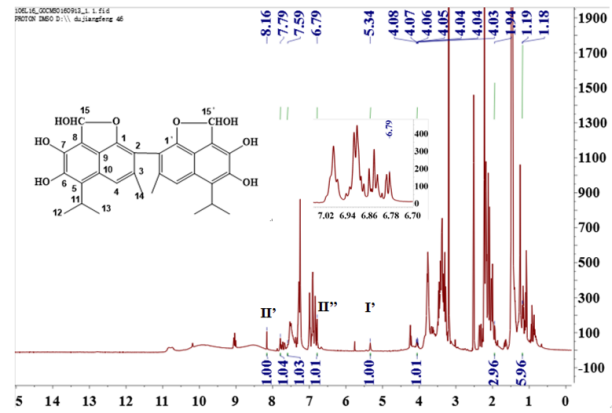


Figure S4. ^1^H NMR spectrum of catalysate.^1^H NMR (400 MHz, DMSO-d_6_) δH 8.16 (s, 15,15’-OH), 7.79(s, H-4,4’), 7.59 (s, 6,6’-OH), 6.79(s, H-15,15’), 5.34(s, 7,7’-OH), 4.05(m, H-11,11’), 1.94(d, H-14,14’), 1.19 (d,H-12,12’,H-13,13’).


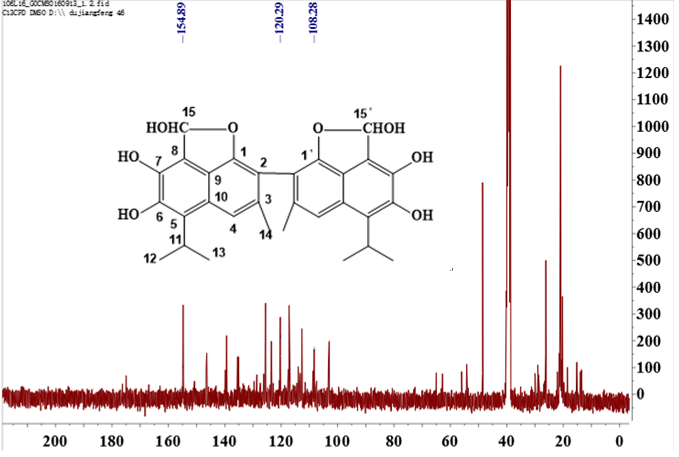


Figure S5. ^13^C NMR spectrum of catalysate.^13^C NMR (400 MHz, DMSO-d_6_) δC 154.89 (C, C-1), 120.29 (C, C-9), 108.28 (C, C-15).


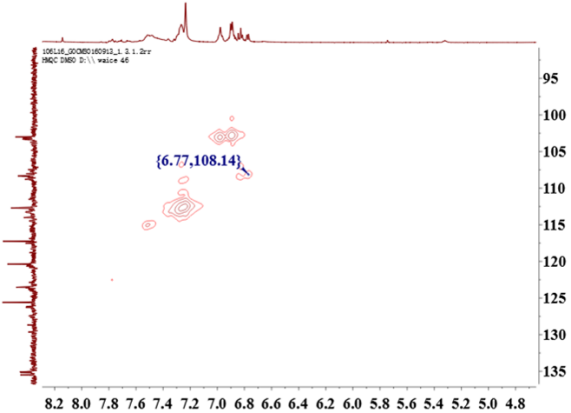


Figure S6. The segment of the ^1^H-^13^C Heteronuclear Multiple Quantum Correlation (HMQC) spectrum of products. The sites shown in the figure correspond to the 6.79(s, H-15,15’) in the figure S4, and 108.28 (C, C-15) in the figure S5. C-15 and H-15 in the furan structure of the analyte molecule are closely correlate.


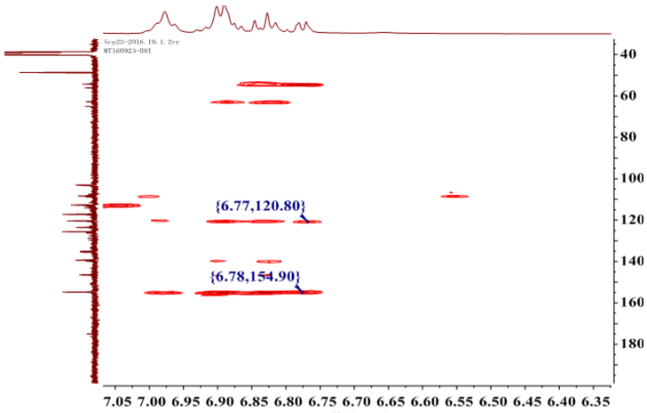


Figure S7. The segment of the ^1^H-^13^C Heteronuclear Multiple Bond Correlation (HMBC) spectrum of product. The two sites shown in the figure correspond to the 6.79(s, H-15,15’) in the figure S4, and 154.89 (C, C-1), 120.29 (C, C-9) in the figure S5, respectively.This shows that C-1, C-9 and H15 are in the same domain but not adjacent.

**
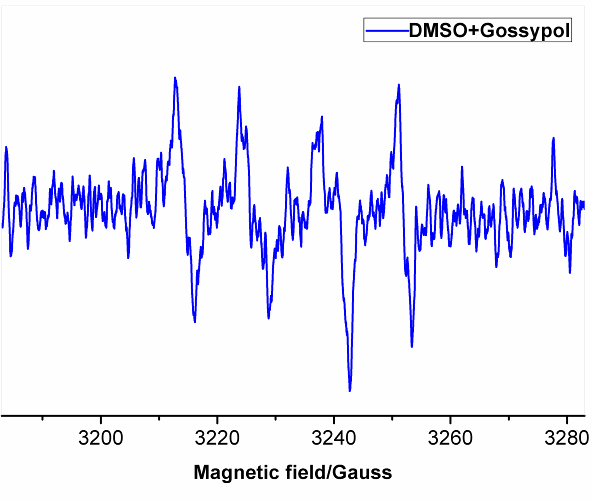
**

Figure S8. EPR spectra of DMPO radical adducts from a sample containing DMSO and gossypol. (a_N_ = 14.70 G, a_Hβ_ = 11.5 G)


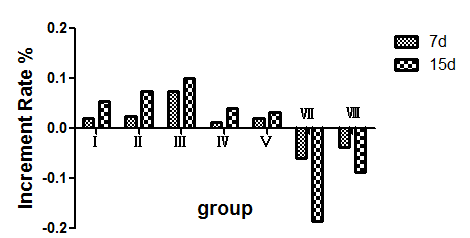


Figure S9. Effects of gossypol and its reaction products on body weight in mice after fed for 7d and 15d. The results represent the mean ± SD of eight animals per group.


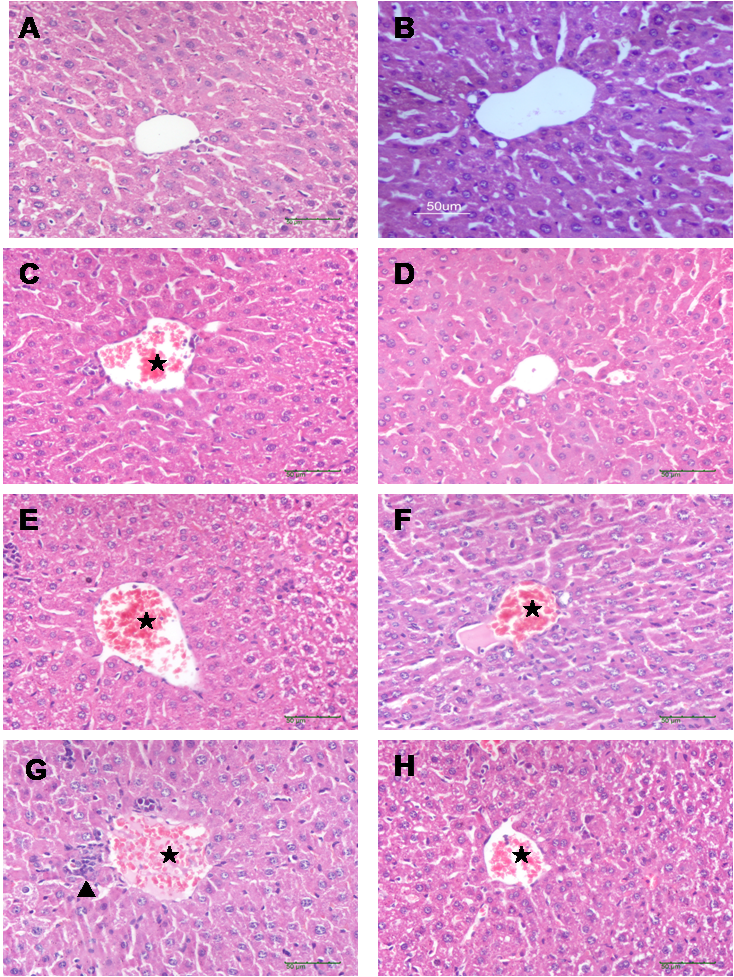


Figure S10. [Hepatotoxicity](http://dict.cn/hepatotoxicity) of gossypol compared to its oxidative products. Liver in mice treated with 10% CMC-Na (A,B), gossypol in dose of 50 **mg/kg** BW (C) , the oxidative products in which the initial gossypol concerntion was 50 **mg/kg** BW (D), gossypol 100 **mg/kg** BW (E), oxidative products 100 **mg/kg** BW (F), gossypol 200 **mg/kg** BW (G) and oxidative products 200 **mg/kg** BW (H), was observed with microscopy (H&E staining , 400 × magnification).


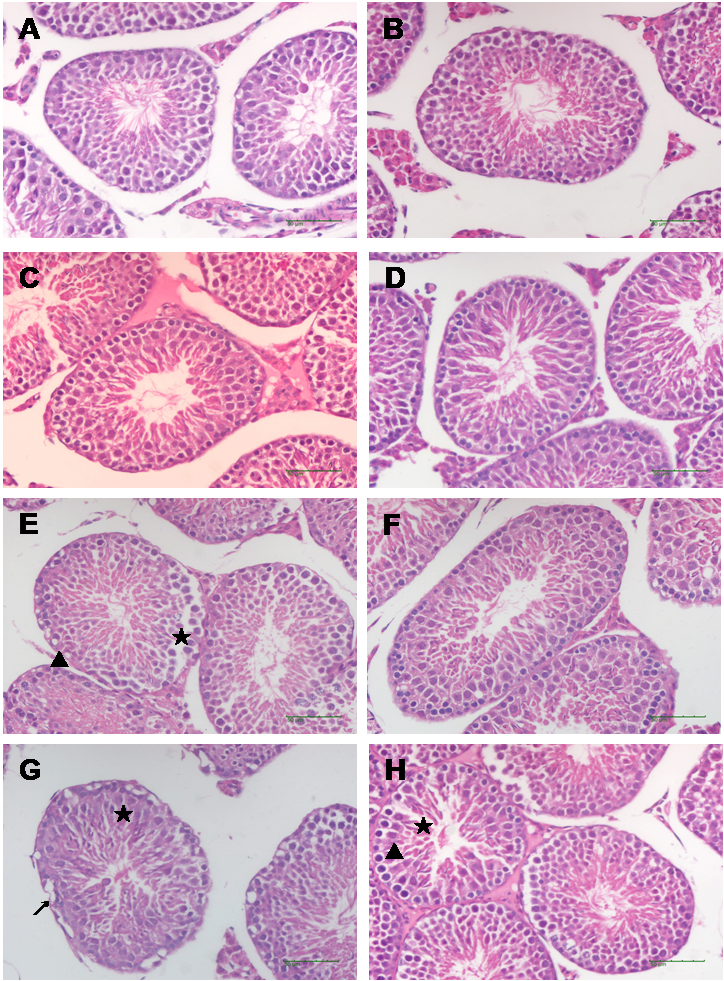


Figure S11. Reproductive toxicity of gossypol compared to its oxidative products. Testis in mice treated with 10% CMC-Na (A,B), gossypol in dose of 50 **mg/kg** BW (C) , the oxidative products in which the initial gossypol concerntion was 50 **mg/kg** BW (D), gossypol 100 **mg/kg** BW (E), oxidative products 100 **mg/kg** BW (F), gossypol 200 **mg/kg** BW (G) and oxidative products 200 **mg/kg** BW (H), was observed with microscopy (H&E staining , 400 × magnification).
